# Evaluating the measures taken to contain a *Candida auris* outbreak in a tertiary care hospital in South India: an outbreak investigational study

**DOI:** 10.1186/s12879-021-06131-6

**Published:** 2021-05-06

**Authors:** Dipu Thareparambil Sathyapalan, Remya Antony, Vrinda Nampoothiri, Anil Kumar, Nandita Shashindran, Jini James, Jisha Thomas, Preetha Prasanna, Akkulath Sangita Sudhir, Jeslyn Mary Philip, Fabia Edathadathil, Binny Prabhu, Sanjeev Singh, Merlin Moni

**Affiliations:** 1 Department of General Medicine and Division of Infectious Diseases, Amrita Institute of Medical Sciences, Kochi, Kerala India; 2 Department of Infection Control and Epidemiology, Amrita Institute of Medical Sciences, Kochi, Kerala India; 3 Department of Microbiology, Amrita Institute of Medical Sciences, Kochi, Kerala India; 4 Department of Geriatrics and General Medicine, Epsom and St Heliers NHS trust, Epsom, UK

**Keywords:** Infection prevention and control, *Candida auris*, Outbreak, Bundle, Implementation, Containment, Hand hygiene, Index case, Multidisciplinary, Low-and middle- income country

## Abstract

**Background:**

*Candida auris* infections are an emerging global threat with poor clinical outcome, high mortality rate, high transmission rate and outbreak potential. The objective of this work is to describe a multidisciplinary approach towards the investigation and containment of a *Candida auris* outbreak and the preventive measures adopted in a resource limited setting.

**Methods:**

This outbreak investigational study was conducted at a 1300-bedded tertiary care academic hospital in South India. The study included 15 adult inpatients with laboratory confirmed *Candida auris* isolates. The outbreak cluster was identified in adult patients admitted from September 2017 to 2019. The system response consisted of a critical alert system for laboratory confirmed *Candida auris* infection and multidisciplinary ‘*Candida auris* care team’ for patient management. The team implemented stringent Infection Prevention and Control (IPC) measures including patient cohorting, standardized therapy and decolonization, staff training, prospective surveillance and introduction of *Candida auris* specific care bundle.

**Results:**

Two outbreak clusters were identified; first cluster occurring between October and November 2017 and the second cluster in May 2018. The cohorts consisted of 7 and 8 *Candida auris* positive patients in the first and second waves of the outbreak respectively with a total survival rate of 93% (14/15). Deployment of containment measures led to gradual decline in the incidence of adult *Candida auris* positive cases and prevented further cluster formation.

**Conclusions:**

The sustained implementation of guideline and evidence-based IPC measures and training of healthcare workers for improving awareness on systematically following standardized protocols of *Candida auris* related IPC practices successfully contained *Candida auris* outbreaks at our hospital. This demonstrates the feasibility of establishing a multidisciplinary model and bundling of practices for preventing *Candida auris* outbreaks in a Low- and Middle-income country.

**Supplementary Information:**

The online version contains supplementary material available at 10.1186/s12879-021-06131-6.

## Background

*Candida auris (C. auris*) infection is an emerging global threat since its first identification in Japan in 2009 [1]*.* Candida species has been identified among 25% of Intensive Care Unit (ICU) patients with central line associated blood stream infections and the prevalence of *C. auris* was estimated to be ranging from 5 to 30% among Candidemia patients [2–4]. The emergence of *C. auris* raises several serious concerns for public health primarily due to its outbreak potential [5]. The outbreaks of *C. auris* described in the USA, UK, and Spain had a high transmission rate [6, 7]. *C. auris* infections are associated with high mortality rate and poor outcomes attributed to high frequency of drug resistance and its tendency to affect immunocompromised patients. The published mortality rate estimated to range from 28 to 78% [8, 9]. Cost of care data associated with *C. auris* infection are scarce, though outbreak control costs were reported to be over £1 million and £58,000/month at an academic tertiary care setting in UK [10]. The identification of *C. auris* requires the updated VITEK-2 yeast identification system or matrix-assisted laser desorption/ ionization time-of-flight (MALDI-TOF) or sequencing the D1-D2 region of the 28 s ribosomal DNA, the availability of which is scarce in developing countries [11]. The common biochemical methods such as analytical profile index strips or the prior version of VITEK 2, often misidentifies *C. auris* as other yeasts (most commonly *Candida haemulonii*, but also *Candida famata, Saccharomyces cerevisiae*, and *Rhodotorula glutinis*) [12].

Implementation of evidence based IPC strategies feasible in low resource settings needs to be explored [13]. *C auris* also differs from other types of *Candida* in its ability to persist on hospital surfaces and spread between patients, although the precise mode of transmission had not yet been identified [2]. The key to *C auris* prevention is strict adherence to infection control measures. Public Health England recommends key IPC practices including isolation of all infected or colonized patients; use of contact precautions in addition to rigorous hand hygiene; screening of close contacts; and a terminal cleaning once infected patients gets discharged [14]. The Director of the Infection control and prevention at the Joint Commission suggests that Infection preventionists (IPs) will help in driving the prevention measures but would be unlikely to be effective as a solo approach. This emphasizes the need of a multidisciplinary approach to tackle the transmission of superbugs [15, 16].

We hereby describe a multidisciplinary approach towards the investigation and containment of *C. auris* outbreak in a resource limited setting, and the comprehensive strategies in the outbreak response comprising of IPC measures, prospective surveillance efforts, healthcare staff training and teamwork employed to contain and prevent further *C. auris* infections across the hospital.

## Methods

### Study design and setting

The current outbreak investigational study was conducted prospectively at Amrita Institute of Medical Sciences (AIMS), Kochi, a 1300 bedded tertiary care academic hospital in South India. The institution is an apex referral centre catering to complex surgical and medical cases and has a robust Antimicrobial Stewardship (AMS) program [17] with a dedicated team for Antifungal Stewardship to ensure appropriateness of antifungal prescriptions. The antifungal stewardship team consisted of an ID physician, clinical pharmacists, microbiologist and a physician with domain expertise in fungal infections. in addition, the institution has a dedicated IPC Team with a total of 6 Infection Control Nurses, who conduct location-based and pathogen-based surveillance of infections. The clinical microbiology lab provides alerts to the IPC team whenever *C. auris* is isolated.

### Study subjects

All the adult inpatients admitted from September 2017 to 2019 with laboratory confirmed *C. auris* isolates were recruited. Pediatrics and neonates were excluded.

### The first wave

**Fig. 1:**
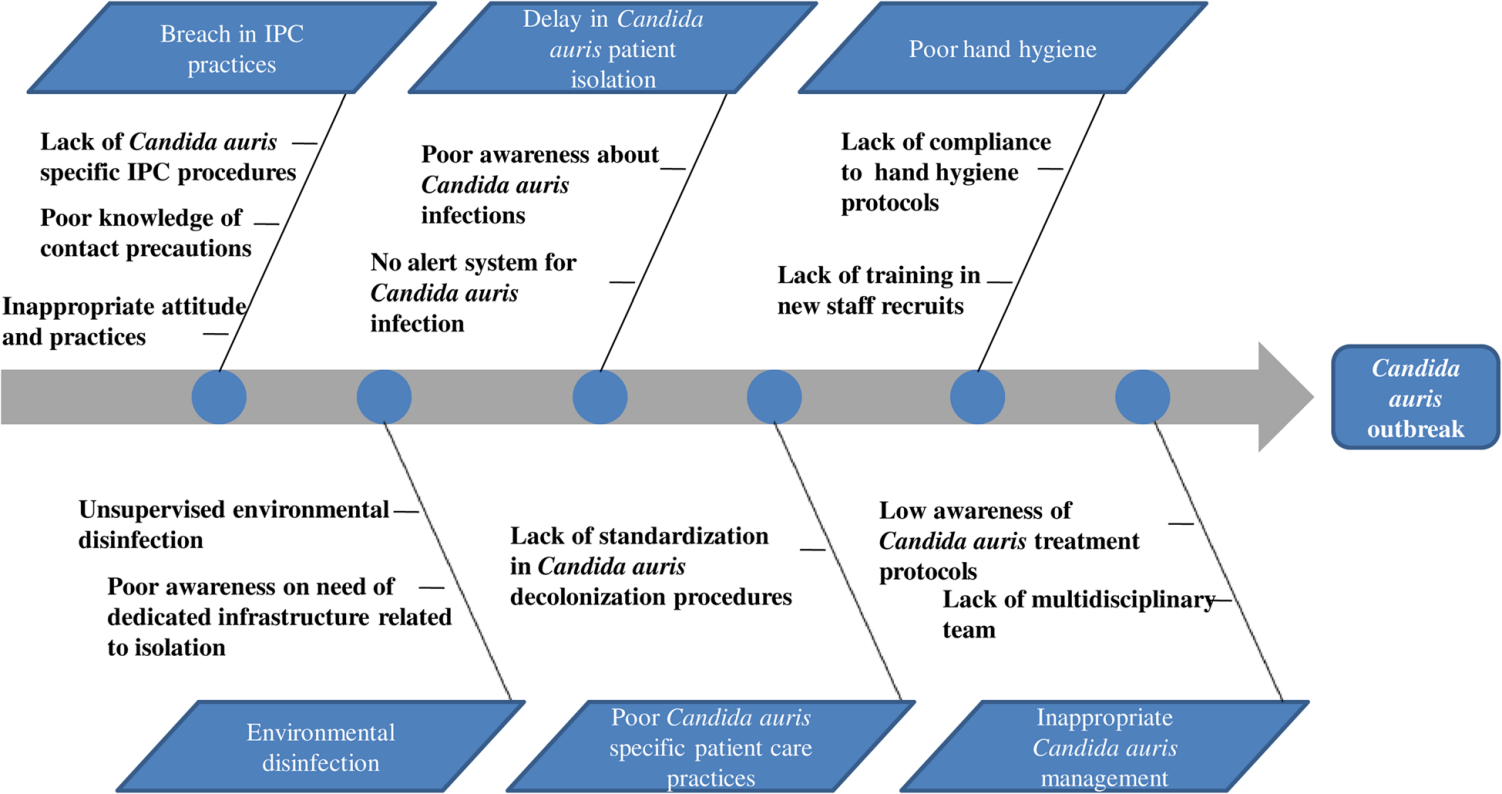
Fishbone diagram depicting factors contributing to *Candida auris* outbreak

Prospective audit of antifungal prescriptions by stewardship team and serial review of microbiological isolate-based weekly surveillance between October and November 2017, revealed a clustering of *C. auris* cases starting at different medical and surgical departments. This was alerted as a potential outbreak on 1st of November 2017. With the identification of the outbreak cluster, a multidisciplinary action team designated as ‘*C. auris* care Team’ was formulated on 3rd November 2017 with Administrative Champion, Infectious disease physicians, AMS team, IPC Team and Microbiologists. Root Cause Analysis of the problem was conducted (Fig. 1) and causal factors in terms of personnel, procedures and environment were explored. An action plan was developed where each team member had specific roles as described in Table 1.

**Table 1:** *C. auris* care team and their responsibilities for *C. auris* outbreak response

Care Team Members	Responsibilities
Clinical Microbiology Team	Issuing an alert to the treating physician, IPC and AMS team when *C.auris* is isolated. The process of outbreak identification is initiated from Microbiology.
IPC team	Routine training on IPC practices to the nursing team.
	Monitoring adherence of IPC practices in the locations where the cases were identified.
	Ensure appropriate isolation or cohorting of patients.
	Ensuring timely and sufficient supply of personal protective equipment (PPE), disinfectant solutions and hand rubs.
	Ensure appropriate cleaning of locations occupied by the patient.
	Education of staff and bystanders regarding IPC practices.
	Prospective surveillance of *C. auris* cases
Infectious Diseases Physician	Decide appropriate therapy and procedures for the patient
	Monitor for clinical improvement and microbiological cure (wherever appropriate)
	Create awareness among primary team and tailor treatment.
	Ensure isolation and proper disinfection
Clinical pharmacist from AMS team	Dedicated member of the team receives critical alert from the Microbiology once *C. auris* is isolated.
	Prepare appropriate treatment regimen and inform the primary team. Follow up for appropriateness of therapy with 5 R’ criteria: Right drug, Right dose, Right frequency, Right duration and Right indication [17].
	Coordinate efforts of all stakeholders in the management of the patient.

The data collected included patient demographics, admission source, comorbid conditions, date of sending cultures, specimen positive for *C. auris* as identified by the updated VITEK 2 system, prior antifungal exposure, treatment received, duration of hospital stay, duration of ICU stay, presence and duration of central venous catheters, procedures (surgery in the last 30 days), clinical and microbiological cure. Incidence of *C.auris* infection and all-cause mortality rate was assessed as primary and secondary outcome respectively.

The definitions used for determining the outbreak and that aided the investigation and its analysis is highlighted in a Table 2.

**Table 2:** Case definitions

Hospital acquired *C. auris* infection	Isolation of *C. auris* from any body fluids obtained from a specimen collected > 48 h after hospital admission.
Prior antifungal exposure	Empirical or prophylactic therapy with antifungals within 30 days prior to the diagnosis of *C. auris* infection.
Clinical cure	Complete resolution of all clinical signs and symptoms of focus of infections pertaining to *C. auris* as evidenced by complete resolution of fever and attainment of hemodynamic stability, if normal before starting treatment.
Microbiological cure	Negative culture or absence of *C. auris* in repeat cultures

### Measures taken by the *C. auris* care team

The team undertook a series of measures to tackle the problem. The first step was confirmation of the presence of the outbreak as identified by a clustering of *C. auris* positive patients over a time span of 1 month which was observed to be greater than the institutional endemic rate [18]. Following the root cause analysis, the team met on a daily basis to formulate containment strategies as per guidelines.

**Fig. 2:**
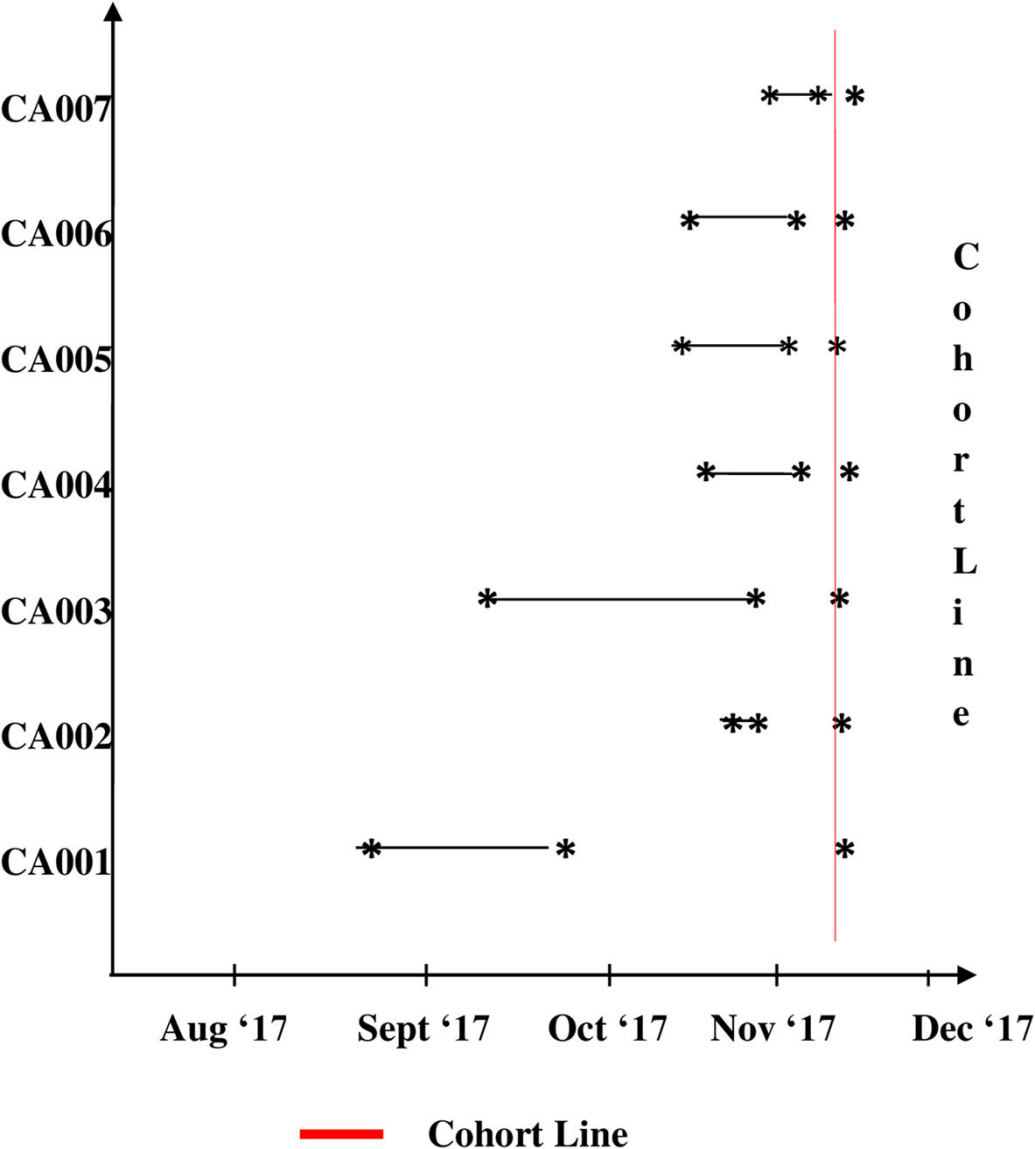
Pictorial representation of length of stay for each patient prior to isolation of *Candida auris*. **NOTE**: The horizontal line shows the duration from Date of Admission (DOA) to Date of Isolation of *Candida auris* (DOI) and the vertical line shows Date of Cohorting (DOC)

The first step was to cohort all the patient cases to a single location. All adult inpatients from ICU and ward (n-7) identified to have any culture positivity for *C. auris* were shifted to a dedicated cohort area for ensuring environmental control since 8th of November 2017. Duration of IP stay prior to cohorting is depicted in Fig. 2. Each patient was kept on 1:1 nursing care.

All staff posted in the designated cohort area, were given training regarding IPC practices specific to *C. auris* using power point and video presentations for sensitization as per the guidelines from the Centers for Disease Control and Prevention (CDC) [19]. It involved standard and contact precautions for all healthcare workers coming in contact with a positive case, re-emphasized the importance of hand hygiene practices, environmental disinfection with 0.5 to 1% of hypochlorite solution and decolonization of the positive cases [20].

A critical alert system through mail for all cases positive for *C. auris* from any sample was created from Microbiology with the aid of the Hospital Information Technology department and sent to all stakeholders. This was for early institution of isolation and infection control measures and ultimately to limit transmission. Once alerted, the care team also initiated patient line listing of the positive cohorted cases*.*

Strict infection control measure was implemented at the cohort area. These measures included:

- Contact precautions with droplet precautions.
- Restriction of number of members visiting the patients.
- A dedicated team was assigned for the clinical care of the *C. auris* patients in the cohort area
- All horizontal measures were strengthened across the hospital by the infection control team.
- Use of PPE (gloves, aprons, and gowns) by healthcare workers.
- A care protocol was developed for all patients admitted to cohort area with positive culture. This included optimizing therapy, chlorhexidine body washes, octenidine wipes [21] and mouth washes and enforcing proper isolation practices with enhanced surface cleaning with chlorhexidine.
- Thorough daily and terminal cleaning and disinfection of patient areas.
- Shared equipments were disinfected before being used in another patient.
- Environmental disinfection was mandated using sodium hypochlorite solution (1 in 10 dilution) in every shift-3 times daily
- Terminal cleaning of the rooms after discharge.

The AMS team prepared and disseminated the protocol for the therapeutic management of *C. auris* patients. This protocol along with active surveillance and Infection Control measures were carried out and incorporated into institutional policies as per the guidelines of CDC [14, 22]. All methods were carried out in accordance with relevant guidelines and regulations.

## Results

### The outbreak

Our study cohort consisted of 7 and 8 *C. auris* positive patients in the first and second waves of the outbreak respectively with a total survival rate of 93% (14/15). Medical departments predominated in both the first and second wave of the outbreak. ICUs constituted 57% (4/7) in the first wave of outbreak while 87.5% (7/8) of the second wave were reported from wards. Mortality rate was observed to be very low at 14% (1/7) in the first cohort and none of the *C. auris* patients expired in the second wave of outbreak (Tables 3 and 5).

**Table 3:** Baseline characteristics and outcome of patients in the first wave of outbreak

Patient ID	CA001	CA002	CA003	CA004	CA005	CA006	CA007
**Age (in years)**	42	52	42	59	30	82	57
**Sex**	Female	Male	Female	Female	Male	Male	Male
**Department**	Stroke medicine	Respiratory medicine	Head and neck surgery and oncology	General medicine	Cardiovascular and Thoracic Surgery	Pulmonary medicine	Endocrinology
**Primary diagnosis**	Stroke	Pneumonia	Malignancy	Pneumonia	Pneumonia	Pneumonia	Skin and soft tissue infection
**Location at the time of Isolation**	Ward	ICU	ICU	Ward	Ward	ICU	ICU
**Prior Antifungal exposure**	yes	no	yes	No (previous admission not known)	yes	Yes	no
**Surgery in the last 30 days**	no	no	yes	no	no	No	yes
**Duration of hospital stay prior to isolation of** ***C. auris (*in days)**	54	2^a^	40	86 (multiple admissions)	14	13	6
**Duration of ICU stay prior to isolation of** ***C. auris (*in days)**	39	1	8	30 (multiple admissions)	8	0	0
**Specimen from which** ***C. auris*** **was isolated**	1.Urine (Foley’s catheter)	Broncho Alveolar Lavage	1.Tracheal aspirate	Pus	Pus	Urine	Tissue
	2.Blood (Central line)		2.Urine				
	3.Urine						
**Clinical cure**	Yes	Yes	Yes	Yes	Yes	No	Yes
**Mortality**	Alive	Alive	Alive	Alive	Alive	Death	Alive

*ICU* Intensive care unit

^a^patient might have acquired the infection from the previous hospital- *C. auris* was isolated in the patient within 2 days of admission in our hospital

### First wave

The 7 adult patients in the first wave were reported to have laboratory confirmed C. auris infection between October and November 2017 with a median age of 52 years (range 30–82 years). Baseline characteristics and outcome of patients have been described in Table 3. The mean duration of hospital stay prior to *C. auris* isolation was 30 days (range 2–86 days) and the ICU stay prior to *C. auris* isolation was 12 days (range 0–39 days).

### Therapeutic management of *C. auris* cases of first cohort

Though treatment was not given to patients with *C. auris* identified from noninvasive sites when there was no evidence of infection, IPC measures including enhanced patient decolonization and environmental disinfection procedures were followed for all these patients. 6 of the 7 patients in the cohort were prescribed echinocandin (micafungin 100 mg once daily) for average treatment duration of 12 days as shown in Table 4. One patient expired before initiation of treatment with Echinocandins. Five patients attained microbiological cure with the exception of 1 patient with *C. auris* colonization who was discharged at request to a local hospital after 6 days of cohorting. The mean duration of isolation at cohort location was 24 days.

**Table 4:** The treatment administered to the *C. auris* patients of the first wave

Patient ID	Treatment	Duration of Echinocandins (in days)	Duration of Amphotericin (in days)
CA001	Micafungin and Amphotericin Bladder wash	29	3
CA002	Micafungin	4	NA
CA003	Micafungin followed by Anidulafungin	10	NA
CA004	Micafungin	9	NA
CA005	Micafungin and Amphotericin	8	5
CA006	Fluconazole (11 days)	0	0
CA007	Micafungin followed by Anidulafungin	12	NA

The cohort location was maintained for 45 days till the last patient was discharged and the incidence of *C. auris* dropped to zero. By December, we observed no new cases apart from a different specimen among the cohort turning out to be positive. Hence after the last patient in the cohort area was discharged, the cohorting was discontinued. However, the active weekly surveillance of *C. auris* positive cases continued.

### The second wave

Continued active surveillance revealed rise in number of *C. auris* cases in May 2018 following which 8 patients reported positive. The index patient in this cluster was a referred case from a peripheral centre, whose cultures at admission turned positive. This was followed by a steep rise of cases within the next 4 weeks. Baseline characteristics, outcome and treatment of our cohort are depicted in Tables 5 and 6 respectively. The patients in second wave had an average duration of hospital stay of 10 days (range 0–23 days) prior to *C. auris* isolation.

**Table 5:** Baseline characteristics and outcome of patients in the second wave

***Patient ID***	*CA018*	*CA019*	*CA020*	*CA021*	*CA022*	*CA023*	*CA024*	*CA00025*
***Age (in years)***	*58*	*33*	*84*	*67*	*66*	*70*	*31*	*52*
***Sex***	*Male*	*Male*	*Male*	*Male*	*Female*	*Male*	*Female*	*Male*
***Department***	*Physical Medicine*	*General medicine*	*General medicine*	*Cardiology*	*General medicine*	*Endocrinology*	*Gastrointestinal surgery*	*Gastroenterology*
***Primary diagnosis***	*Stroke*	*Sepsis, Pneumonia*	*Skin and soft tissue infection*	*Complete Heart block*	*Otomastoiditis*	*Skin and soft tissue infection*	*Malignancy*	*Liver cirrhosis*
***Location at the time of Isolation***	*ICU*	*ward*	*ward*	*ward*	*ward*	*ward*	*ward*	*Ward*
***Prior Antifungal exposure***	*NA*	*yes*	*Yes*	*no*	*yes*	*no*	*no*	*Yes*
***Surgery in the last 30 days***	*yes*	*no*	*Yes*	*yes*	*no*	*no*	*yes*	*No*
***Duration of Hospital stay prior to isolation of C. auris (in days)***	*0*	*9*	*18*	*4*	*10*	*4*	*15*	*23*
***Duration of ICU stay prior to isolation of C. auris (in days)***	*0*	*8*	*3*	*0*	*0*	*0*	*12*	*6*
***Specimen from which C. auris was isolated***	Urine	Urine	Urine	Tissue	Urine	Nasal swab	Tissue	Broncho alveolar lavage
***Clinical cure***	*Yes*	*Yes*	*Yes*	*Yes*	*Yes*	*Yes*	*Yes*	*Yes*
***Mortality***	*Alive*	*Alive*	*Alive*	*Alive*	*Alive*	*Alive*	*Alive*	*Alive*

**Table 6:** The treatment administered to the *C. auris* patients of the second wave

Patient ID	Treatment	Comments
CA0018	No systemic antifungals	Urine colonisation-Source Control done
CA0019	No systemic antifungals	Urine colonisation-Source Control done
CA0020	No systemic antifungals	Tissue colonisation-Source Control done
CA0021	No systemic antifungals	Tissue colonisation-Source Control done
CA0022	Anidulafungin	Osteomyelitis
CA0023	No systemic antifungals	Tissue colonisation- Source Control done
CA0024	No systemic antifungals	Wound colonisation- Source Control done
CA0025	No systemic antifungals	Urine clonisation- Source Control done

All cases were identified by the candida care team and isolated with strict contact and droplet precautions. The primary treating team was notified regarding the culture positivity and treatment was optimized by the Infectious Diseases physicians in the AMS team. The patients were isolated for the entire inpatient stay with 1:1 nursing and infection control measures. A bundle of care checklist was created by the AMS team enlisting the CDC guidelines to be followed for *C. auris* patients [see Additional file 1]. The bundle components included twice daily body bath with chlorhexidine, source control, enhanced surface cleaning and education of patient, bystander and treating team. This was filed within the flagged patient file.

The containment measures and infection control protocols were standardized across the institution with the sensitization of the primary care team. Information pamphlets with the IPC measures and standard protocols to be followed while handling *C. auris* patients were given to the designated clinical staff and ward ancillary staff taking care of these patients along with one-to-one awareness classes and bedside training [see Additional file 2].

**Fig. 3:**
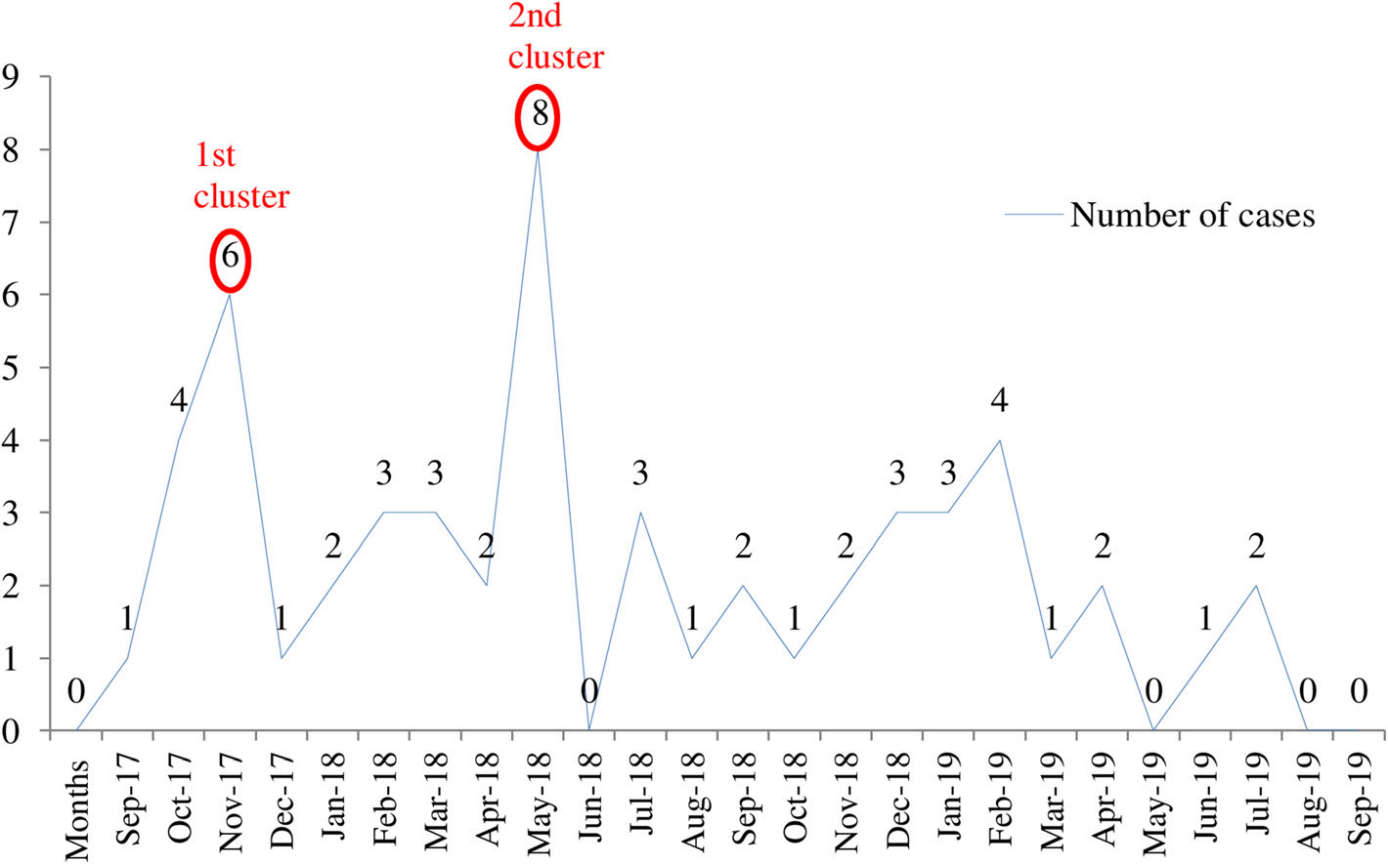
The incidence of Candida auris in the centre from September 2017 to September 2019

By 1st September 2019, active surveillance with sustained measures, incidence of adult *C. auris* positive cases gradually decreased and reached endemic rates (Fig. 3).

## Discussion

We report *C. auris* specific care bundles and IPC measures adopted at our Low- and Middle-Income Country based healthcare center that led to the successful containment of two outbreak waves of *C. auris*. The sustained deployment of stringent IPC measures and clinical care bundle undertaken during the second wave of *C. auris* outbreak not only flattened the curve of *C. auris* incidence, but also prevented further outbreak waves at the hospital (Fig. 2).

The first wave of outbreak triggered a comprehensive containment plan of IPC procedures that focused on cohorting of *C. auris* positive patient cases in addition to generating awareness among primary clinical care team on the importance of *C. auris* infections, its risks and management. Even though surveillance activities were continued, a second outbreak was encountered at the institute which could be probably due to lack of sustained efforts in maintaining the IPC practices. The *C. auris* specific bundle implemented as a response to second outbreak wave stressed on standardized practices for patient decolonization at the location of *C. auris* identification and environmental cleaning as *C. auris* is associated with transmission through surface contaminations [23]. Targeted efforts towards containment were adopted with a multidisciplinary approach encompassing IPC, infectious diseases physicians, antimicrobial stewardship, clinical microbiologists, clinical pharmacists, nursing and primary care team of the *C. auris* infected patient. Our patient cohort included both ICUs and wards as locations at the time of *C. auris* isolation, unlike previous outbreak investigations primarily citing ICUs as major locale of infections attributing to the use of invasive devices, prolonged patient stay and numerous medical procedures [24]. Middle aged and elderly patients predominated in the outbreak waves as *C. auris* has been observed to afflict vulnerably aged populations. Mortality rates were consistently low with only 14.2% patients expired in the first wave and none in the second wave. *C. auris* candidemia patients were previously reported to have a mortality of 41.9% in an Indian ICU based study [25]. Echinocandins are the first line agents for treatment of *C. auris* based on existing evidence [7]. Micafungin was therefore used as the major antifungal drug to treat all *C. auris* positive patients of our first outbreak cohort except a single case, for which fluconazole was used. Patients in our second cohort mostly had asymptomatic colonisation for which stringent IPC measures were taken except for a single patient for whom anidulafungin was given. The possible explanation of the occurrence of a second wave inspite of the IPC measures implemented could have been due to the inability of *C auris* specific training to keep pace with the high turnover rate of care providers and potential import of index cases due to the institution being an apex tertiary care referral centre.

The management of *C. auris* in developing countries are impacted by poor outcomes on account of inadequate IPC practices permeating the spread of infections, non-availability of advanced diagnostic tests, lack of the recommended drug echinocandin and paucity of robust data on *C. auris* infection and antifungal susceptibility rates [13]. Though the updated VITEK automated identification system was available since 2017 at our institution for accurate identification of *C. auris* and guide management, *C. auris* genome sequencing to understand azole and echinocandin resistance association of geographic clades and clonal features was not an affordable strategy in our study. A novel clonal strain of *C. auris* was reported previously from healthcare centers at a single locale in India isolated over a span of 2 years. This clone was identified to be genotypically different from isolates from South Korea and Japan [26]. The distinct clonal origin was subsequently reported for a total 26 *C. auris* isolates all over India including a single isolate from our institution [27, 28].

Nonetheless, accurate identification of *C. auris* isolates is still considered as a diagnostic challenge in India, due to which pragmatic solutions are recommended for addressing the infection [29]. The CDC IPC recommendations for *C. auris* transmission- based precautions calls for appropriate communication of *C. auris* status during patient transfer to healthcare centers, an unfeasible option in countries of squalid health infrastructure, poor data sharing platforms and diagnostic capabilities [19]. This warrants the need of imparting awareness on *C. auris* infections and IPC measures for healthcare workers to sensitize them towards effective management and initiate surveillance measures. The community-based impact of *C. auris* infections should also be addressed which has an unexplored public health perspective.

### Limitations

This is an outbreak response from a single center and the extent of spread in the community has not been determined. Pediatrics and neonates were excluded in this study.

## Conclusions

The sustained and stringent implementation of guideline and evidence-based IPC measures and training of healthcare workers for improving awareness on systematically following standardized protocols of *C. auris* related IPC practices successfully contained two outbreak waves of *C. auris* infections at our hospital. The outbreaks alerted us that the emerging etiological agent will stay in the healthcare for a prolonged period, prompting us to continue the precautions for a longer period and to be vigilant in preventing further outbreaks and clusters. Through a multimodal strategy including prompt identification, surveillance, reporting, strict infection control measures and appropriate antifungal treatment, we can mitigate the spread and prevent the reporting of new *C. auris* positive cases.

## Supplementary Information

**Additional file 1.**

**Additional file 2.**

## Data Availability

The datasets used and/or analyzed during the current study are available from the corresponding author on reasonable request.

## Abbreviations

*C.auris*: *Candida auris*
IPC: Infection Prevention and Control
ICU: Intensive Care Unit
MALDI-TOF: Matrix-assisted laser desorption/ ionization time-of-flight
IPs: Infection preventionists
AMS: Antimicrobial Stewardship
PPE: Personal protective equipment
CDC: Centers for Disease Control and Prevention
