# Supplementary material for: Evaluating the measures taken to contain a Candida auris outbreak in a tertiary care hospital in South India: an outbreak investigational study
Source: BMC Infect Dis. 2021 May 6;21:425. doi: 10.1186/s12879-021-06131-6 (PMC8101254; doi:10.1186/s12879-021-06131-6)
Supplement: Supplementary file 1 — Additional file 1. [file 12879_2021_6131_MOESM1_ESM.pdf]

Additional file 1

***Candida auris* checklist**

Sl. No	**Measures to be executed**	**Yes**	**No**
1.	Isolate the patient with 1:1 nursing and follow standard and contact precautions		
	Hand hygiene		
	Gloves, Gown, Mask		
	Eye protection or face shield (in case of sputum candida positive)		
2.	Patient decolonisation using Chlorhexidine or Octenicin body wash		
3.	Environmental disinfection using 0.5% to 1% Sodium hypochlorite		
4.	Identification of colonisation / invasive infection		
5.	In case of fungemia:		
	Appropriate selection of Antifungals		
	Repeat blood culture after 48hours. If positive rule out endocarditis using TEE/TTE		
	Antifungal administered for 14days after documented negative blood cultures		

6.	Source control		
7.	Terminal sterilization of equipments using 0.5% to 1% sodium hypochlorite		
8.	In case of surgery, infected patients should be the last to be taken for surgery on a given day.		
