# Supplementary material for: Evaluating the measures taken to contain a Candida auris outbreak in a tertiary care hospital in South India: an outbreak investigational study
Source: BMC Infect Dis. 2021 May 6;21:425. doi: 10.1186/s12879-021-06131-6 (PMC8101254; doi:10.1186/s12879-021-06131-6)
Supplement: Supplementary file 2 — Additional file 2. [file 12879_2021_6131_MOESM2_ESM.pdf]

Additional file 2

***Candida auris* specific IPC activities**

Information pamphlet for healthcare workers

Isolation

- Single-patient room or in rooms with other patients with *C. auris.*

Duration of Infection control precautions

- CDC currently recommends continuing Contact Precautions until the patient is discharged from hospital.

Enviornmental disinfection

- 1 in 10 dilution sodium hypochlorite solution / wipes
- In every shift-3 times daily
- When possible after discharge /transfer-terminal cleaning of the room with fogging.

Decolonisation

- Adult to be wiped with 2% Chlorhexidine
- Pediatric patients to be wiped with Octenicin.
- At least change linen once daily unless soiled.
- Must be disinfected separately.

<u>Treatment</u>

<u>Adults and children ≥ 2 months of age</u>

**Echinocandin Drug**	**Adult dosing**	**Pediatric dosing**
Micafungin	100 mg IV daily	2mg/kg/day IV with option to increase to<br>4mg/kg/day IV in children 40 kg
Anidulafungin	loading dose 200 mg IV,<br>then 100 mg IV daily	not approved for use in children
Caspofungin	loading dose 70 mg IV,<br>then 50 mg IV daily	loading dose 70mg/m^2/day IV, then<br>50mg/m^2/day IV<br>(based on body surface area)

Switching to a liposomal amphotericin B (5 mg/kg daily) could be considered if the patient is clinically unresponsive to echinocandin treatment or has persistent fungemia for >5 days.

<u>Neonates and infants <2 months of age</u>

**Echinocandin Drug**	**Neonatal dosing**
Caspofungin	25 mg/m^2/day IV<br>(based on body surface area)
Micafungin	10mg/kg/day IV
